# From Challenges to Innovations: Expert Insights in Pediatric Healthcare Design

**DOI:** 10.1177/19375867251353733

**Published:** 2025-07-24

**Authors:** Haripriya Sathyanarayanan, Luisa Caldas

**Affiliations:** 1 1438 University of California, Berkeley, USA; 2 Berkeley XR Lab, 1438 University of California, Berkeley, USA; 3 Lawrence Berkeley National Laboratory, USA

**Keywords:** pediatric healthcare design, patient-centered design, patient experience, emerging technologies, expert insights, virtual reality, design challenges, flexibility

## Abstract

**Background:** Pediatric healthcare design increasingly addresses clinical and psychosocial needs, highlighting the role of spatial, technological, and environmental factors in supporting patient well-being. Emerging technologies, such as virtual reality (VR) and artificial intelligence (AI), show potential for improving patient engagement and healthcare operations, though adoption remains challenged by privacy, ethical, and implementation barriers. **Objectives:** This study explores current trends, barriers, and future directions in pediatric healthcare design, focusing on patient-centered care, adaptability, emerging technologies, and participatory design strategies. **Methods:** Fifteen experts—including architects, pediatric nurses, child life specialists, and healthcare administrators—were interviewed via Zoom using a semi-structured format. Purposive and snowball sampling facilitated recruitment, and thematic analysis was conducted to extract key themes. **Results:** Experts highlighted the evolution of family-centered and culturally responsive design, emphasizing the need for privacy-conscious, adaptable spaces that support neurodivergent children and behavioral health needs. Biophilic and sustainable design were seen as essential for creating calming environments and supporting long-term well-being. While VR and AI offer promise in enhancing patient experiences and operational efficiency, privacy concerns, costs, and regulatory concerns remain key barriers. Infection control measures, while critical, were seen as potentially conflicting with patient-centered and socially supportive environments. Interdisciplinary collaboration and participatory design methods were underscored as key to addressing complex design challenges. **Conclusions:** Future pediatric healthcare design will prioritize flexibility, inclusivity, and a public health approach, balancing clinical safety with emotional and psychological well-being. Ongoing interdisciplinary collaboration and adaptive design strategies will be critical in creating environments that enhance patient experience, caregiver support, and healthcare efficiency.

## Introduction

Pediatric healthcare design has shifted from a focus on functionality toward a more holistic approach that prioritizes the well-being of young patients and their families. The physical environment plays a critical role in shaping health outcomes, with evidence demonstrating that well-designed spaces—incorporating natural light, calming colors, and access to nature—can reduce stress, improve clinical outcomes, and shorten hospital stays (Acosta et al., 2017; Bevan et al., 2019; Felippe et al., 2017; Fricke et al., 2019; Nourmusavi Nasab et al., 2020). This evolution reflects broader trends emphasizing patient- and family-centered care, particularly in pediatric settings, where family engagement is fundamental to the healing process (Flynn et al., 2019; Hill et al., 2018; Williams et al., 2021).

In recent years, design strategies for pediatric healthcare have emphasized creating adaptable, patient-centered spaces that integrate therapeutic elements to enhance engagement and comfort (Bogaert, 2022; Kim, 2023; Payam et al., 2023; Seniwati et al., 2023; Smith, 2018; Vollmer & Koppen, 2021). Biophilic and sustainable principles, incorporating natural materials, greenery, and daylight, have been shown to promote psychological and physiological well-being (Kellert et al., 2011). Concurrently, emerging technologies such as virtual reality (VR) and artificial intelligence (AI) are transforming healthcare design by enabling immersive visualizations, therapeutic applications, and operational efficiencies (Bailey & Bailenson, 2017; Eijlers et al., 2019; Pallavicini & Bouchard, 2019; Topol, 2019). Biometrics, capturing physiological and emotional responses to environmental stimuli, provides data-driven insights for design strategies (Bower et al., 2019; Kim & Kim, 2022; Valentine, 2024). Despite the potential, these technologies pose challenges related to privacy, ethics, and cost (Chen et al., 2020; Montreuil et al., 2021).

Systemic barriers, such as infection control requirements and operational inefficiencies, also limit the implementation of patient-centered and participatory approaches in healthcare design (Montreuil et al., 2021; Sathyanarayanan & Caldas, 2022). Participatory Design, also known as User-Centered Design or Co-Design, is a collaborative approach that involves stakeholders—including patients, families, and healthcare professionals—in decision-making processes (Robertson & Simonsen, 2012). By incorporating user perspectives, this method ensures spaces address functional, emotional, and cultural needs. In pediatric healthcare, participatory approaches can create environments that reflect the unique needs of children and their families, fostering engagement and well-being.

Designing for neurodivergent children and those with behavioral health challenges requires sensory-friendly, inclusive environments (Williams et al., 2023). Spaces must accommodate diverse needs while fostering comfort and belonging, incorporating design elements such as noise control, tactile-friendly surfaces, and biophilic features to reduce stress and promote recovery (Higuera-Trujillo et al., 2020; Lauwers et al., 2024). Addressing these multifaceted demands requires interdisciplinary collaboration among designers, clinicians, and patient advocates. Culturally sensitive features—such as multilingual signage, storytelling spaces, and family-oriented areas—can further enhance the patient experience (Kaihlanen et al., 2019).

This study explores how pediatric healthcare design can address these challenges and leverage emerging technologies to advance patient outcomes and family engagement. By synthesizing insights from experts, the research aims to fill critical knowledge gaps and provide actionable recommendations for the creation of adaptable, inclusive, and technologically integrated healthcare spaces. Specifically, the study addresses the following research questions:

1. How do experts describe current trends and the evolution of patient-centered care in pediatric healthcare environments?
2. What barriers do professionals identify in implementing patient-centered and participatory design strategies in pediatric healthcare environments?
3. How are emerging technologies, particularly immersive technologies, being utilized in the design process for pediatric healthcare environments, and what impacts do experts anticipate from their integration?
4. What are the anticipated future directions for pediatric healthcare environments, and how are these expected to influence design practices and enhance patient and family engagement?

## Methods

This qualitative study explored expert perspectives on trends, barriers, and opportunities in pediatric healthcare design using purposive and snowball sampling (Palinkas et al., 2015). Participants were selected based on direct experience in designing, managing, or researching pediatric healthcare environments. This phase focused on expert insights, with complementary studies addressing patient and caregiver perspectives to ensure a comprehensive research framework.

Fifteen experts were recruited based on their recognized expertise and direct experience in pediatric healthcare design. Five others did not respond to the invitation, an architect specializing in pediatric healthcare design, design researchers, and a healthcare owner. Physicians were initially considered, but were excluded due to scheduling challenges and limited availability during the COVID-19 period. Selection criteria prioritized diverse professional roles, including pediatric nurses, architects, healthcare administrators, child life specialists, psychologists, and researchers, with substantial experience in practice, research, or policy. Participants were identified through analysis of published works, professional networking platforms such as LinkedIn, direct outreach via email, and observations from industry webinars and conference presentations. Snowball sampling was subsequently employed to expand participation and capture a wider range of perspectives. No prior professional affiliations or collaborations existed between the study authors and the interviewees before recruitment. Table 1 outlines participant roles, with several experts possessing dual expertise: P1–P4 were Pediatric Registered Nurses and researchers, P5 and P11–P12 combined architectural and research expertise, and P10 was a Psychologist and Pediatric Researcher.

**Table 1.** Participant Characteristics.

Participant ID	Role/background
P1–P4	Pediatric Registered Nurse & Researcher
P5	Architect & Researcher
P6–P9	Pediatric Healthcare Architect
P10	Psychologist & Pediatric Researcher
P11–P12	Pediatric Healthcare Architect & Researcher
P13, P15	Hospital Owner
P14	Child Life Specialist

The study, conducted between June 2020 and June 2021, invited experts via personalized emails detailing the study's scope and significance. One-on-one interviews (∼60 minutes) were conducted on a secure Zoom platform using a semi-structured interview protocol (see Supplemental Material). Topics included pediatric healthcare design trends, patient-centered strategies, integration of emerging technologies (e.g., VR and AI), and barriers to implementation. All interviews were audio and video recorded and transcribed using Otter.ail. The lead researcher reviewed and corrected transcripts for accuracy before proceeding with thematic analysis.

**Figure 1.**
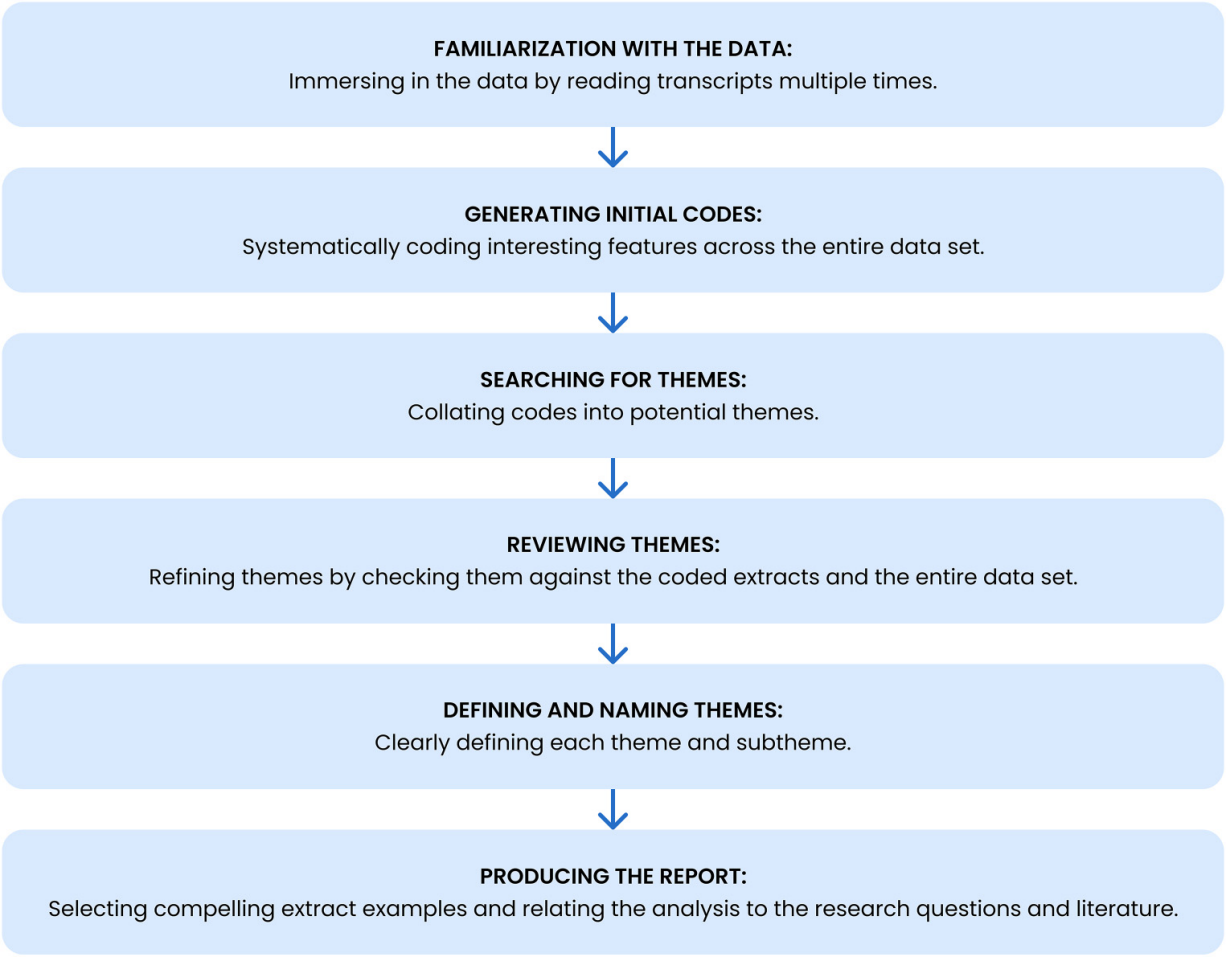
Thematic analysis framework based on Braun & Clarke's six-phase approach (adapted from Braun & Clarke, 2006).

Data were analyzed using Braun and Clarke's (2006) six-phase framework for thematic analysis, which included familiarization, coding, theme identification, and iterative refinement (Figure 1). MAXQDA, a qualitative data analysis software program (VERBI Software, 2021), was initially used for early coding and codebook development. Subsequently, the dataset was manually coded in Excel. The lead researcher and a research assistant independently coded a subset of interviews, generating approximately 200 codes that were reconciled into broader categories through discussion. A collaboratively developed codebook guided the subsequent analysis, which the lead researcher applied iteratively to the full dataset from 2020 to 2021. In 2024, the dataset was revisited to refine themes for publication, aligned with the original research questions.

Examples of identified themes included “Design and Trends” (subthemes: Patient Room Design, Private vs. Semi-Private Rooms) and “Technology Integration” (subthemes: VR for Pain Management, AI in Clinical Decision-Making) (see Table 2 for a full list of themes and subthemes). The final analysis identified 55 themes and 266 subthemes. To ensure accuracy, all themes and subthemes were systematically cross-checked against raw transcripts by the lead researcher through an iterative process, ensuring alignment with participant's original narratives.

**Table 2.** Summary of Themes and Subthemes Addressing Research Questions on Pediatric Healthcare Design.

Research question	Theme	Subtheme
RQ1: Evolution of Patient-Centered Care in Pediatric Healthcare Environments and Current Trends Includes themes that focus on the innovative aspects, and evolution in design practices.	Care Models	Family-Centered Care, Feedback Incorporation
	Design Evolution and Adaptation	Adaptive & Flexible Design, Functional Obsolescence
	Design Innovations	Innovative Patient Room Layouts, Beyond Esthetics, Inclusive Design
	Sustainable Design Practices	Daylighting, Natural Materials, Natural Ventilation, Energy Efficiency
	Biophilic Design	Integration with Nature, Access to Green Spaces
	Behavioral and Mental Health	Behavioral Health Needs, Emotional Support
	Adaptability	Design Flexibility, Versatile Use of Space
RQ2: Barriers and Challenges in Implementing Design Strategies and Participatory Methods. Includes themes that address obstacles, constraints, and practical challenges in the design and implementation process.	Operational Efficiency	Staff Efficiency, Perception of Care, Same-handed rooms, Automated Guided Vehicles
	Patient and Staff Interaction	Design Impact on Staff Efficiency, Staff Interaction
	Infection Control	Air Quality and Infection Control, Visitor Management
	Privacy	Privacy Needs of Children, Acoustic and Visual Privacy
	Neurodivergent Considerations	Design for Neurodivergent Individuals, Sensory Integration
	Sensory Considerations	Multisensory Environment, Noise Reduction, Sensory Elements
	Engagement and Participatory Design	Child and Family Engagement, Community Involvement, Participatory and Collaborative Design, Responsiveness to Feedback, Metrics and Evaluation
	Family Engagement	Role of Family in Design, Sibling Considerations
RQ3: Integration of Emerging Technologies in Design Includes themes that highlight the incorporation of new and emerging technologies in pediatric healthcare design	Technology Integration	Communication, VR for Design Decisions, VR for Pain Management, Positive Outcomes
	Advanced Patient Care	AI in Medical Records, Technological Connection, Telemedicine, Tailored Treatment
RQ4: Future Directions in Pediatric Healthcare Design Includes themes that explore potential future trends and innovations that could shape the field	Future Directions	Public Health Approach, Flexibility, Healthcare Evolution
	Feedback and Evaluation	Post-Occupancy Evaluations, Personalized Feedback
	Cultural and Emotional Factors in Design	Cultural Relevance in Design, Cultural Sensitivity, Emotional Engagement with Design Elements
	Emotional and Social Support	Storytelling as Healing, Importance of Social Interaction, Peer Interaction, Emotional Engagement
	Patient Experience	Control and Autonomy, Patient Experience Data

Participant responses were categorized by professional role to facilitate a structured synthesis of perspectives, allowing for a comparative understanding of role-specific priorities and challenges in pediatric healthcare design. Themes and subthemes were analyzed to identify unique and overlapping viewpoints across groups, ensuring alignment with RQ1 (trends), RQ2 (barriers), RQ3 (technology integration), and RQ4 (future directions). The lead researcher synthesized role-based responses, identifying both profession-specific insights and interdisciplinary overlaps. This structured approach strengthened the robustness of findings while maintaining alignment with the research questions.

Although inter-coder reliability was not calculated, credibility was ensured through collaborative codebook development, iterative coding, and revisiting data. Reflexivity—the process of critically examining one's own role, assumptions, and potential biases—was an integral part of the analysis process, with detailed documentation maintained to ensure transparency and consistency (Blair, 2015).

Ethical approval was obtained from the Committee for the Protection of Human Subjects at University of California, Berkeley (CPHS Protocol #2020-04-13200). Informed consent was obtained, and confidentiality was maintained through anonymization and secure data storage.

## Results

**Figure 2.**
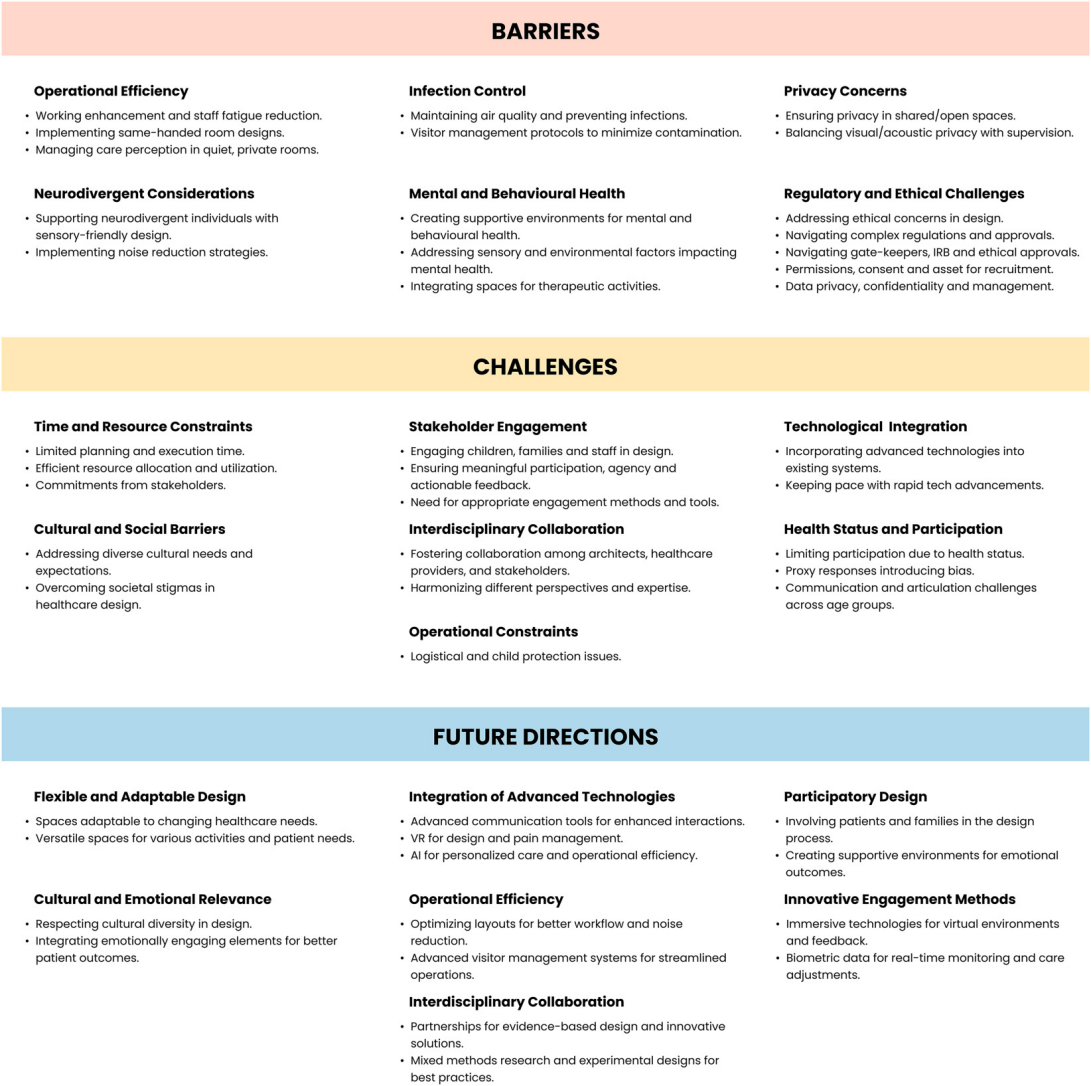
Barriers, challenges, and future directions in pediatric healthcare design.

**Figure 3.**
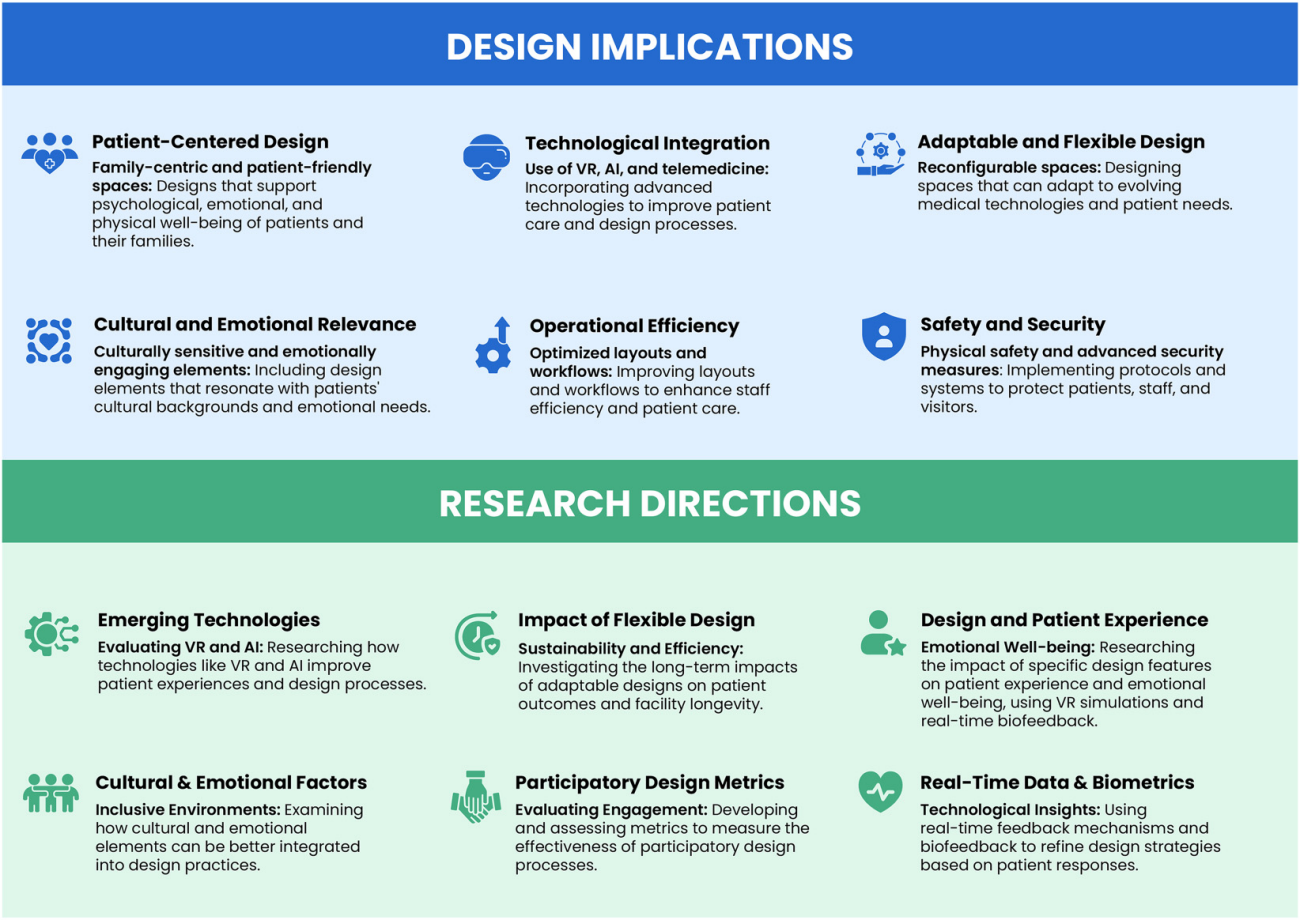
Design implications and research directions in pediatric healthcare environments.

The expert interviews revealed key themes and subthemes in pediatric healthcare design, aligned with the four research questions. These findings included insights shared across roles and profession-specific priorities, reflecting the diverse expertise of participants. Figures 2 and 3 illustrate these trends, barriers, technology integrations, and future directions (Table 2). Table 3 presents representative quotes that deepen understanding of these themes.

**Table 3.** Detailed Themes, Subthemes, and Representative Quotes.

Theme	Subtheme	Quote
*RQ1: Evolution of Patient-Centered Care in Pediatric Healthcare Environments and Current Trends*		
Care Models	Family-Centered Care	“Patient-centered care was a big driver, and in pediatric hospitals, it quickly becomes family-focused care.” [P13] “When a child is sick, the parents are sick. Therefore, environments must be developed to comfort the family.” [P15]
	Feedback Incorporation	“We did a lot of learning, and it allowed us to understand the needs and challenges right away.” [P15]
Design Evolution and Adaptation	Adaptive Reuse and Flexibility	“We need spaces that can be reconfigured for different uses without significant structural changes.” [P3] “Creating adaptable spaces is critical for the longevity and functionality of healthcare facilities.” [P6]
	Functional Obsolescence	“Designing with obsolescence in mind allows us to anticipate future needs and potential technological advancements, avoiding costly renovations.” [P7]
Design Innovations	Innovative Patient Room Layouts	“We've seen a shift towards more family-centered room designs that allow for greater privacy and better support for both patients and their families.” [P10]
	Beyond Esthetics	“Design should serve a therapeutic purpose, with elements like soothing colors and nature-inspired motifs enhancing the healing process.” [P12].
	Inclusive Design	“Inclusive design means creating environments that everyone can use and feel comfortable in.” [P5]
Sustainable Design Practices	Natural Materials and Daylighting	“Incorporating natural materials and ensuring abundant daylight creates a more welcoming and healing environment for patients.” [P5]
	Natural Ventilation	“Natural ventilation is important for maintaining good air quality, which is essential for patient health and comfort.” [P6]
Biophilic Design	Integration with nature	“Biophilic design, including indoor plants and views of nature, reduces stress and improves patient outcomes.” [P8]
	Access to green Spaces	“Biophilic design is more than just adding plants; it's about creating a holistic connection with nature that promotes well-being.” [P9]
Behavioral and Mental Health	Mental Health Needs	“Designing spaces that cater to the mental and behavioral health of children can significantly improve their overall well-being.” [P14]
	Emotional Support	“Spaces that provide emotional support through comforting design elements can help reduce anxiety and promote healing.” [P2]
Adaptability	Design Flexibility	“Flexible designs allow healthcare environments to evolve with new technologies and changing patient needs.” [P3]
	Versatile use of space	“Designing multipurpose areas that can serve different functions as needed is crucial for future-proofing healthcare facilities.” [P6]
*RQ2: Barriers and Challenges in Implementing Design Strategies and Participatory Methods*		
Operational Efficiency	Layout Optimization	“Optimizing the layout to minimize staff travel time between patient rooms can significantly improve efficiency.” [P5]
Privacy Concerns	Balancing Privacy and Supervision	“Children need spaces where they can feel secure and private for their emotional well-being.” [P2]
	Perception of Care in Quiet Spaces	“Brand new units with all private rooms where everything's really quiet and everything's hidden away, can make it seem like nobody's here, nobody's caring for me…” [P5]
	Acoustic and Visual Privacy	“Balancing privacy with the need for supervision is tricky but critical in pediatric settings.” [P11]
Neurodivergent Considerations	Sensory-Friendly Design	“Spaces that accommodate sensory sensitivities help create a more comfortable and accessible environment for neurodivergent patients.” [P11]
Engagement and Participatory Design	Patient and Family Involvement	“Engaging patients and families in design decisions leads to spaces that better meet their needs.” [P10]
	Community Involvement	“Structured family advisory councils provide valuable insights from families with hospital experience.” [P15]
*RQ3: Integration of Emerging Technologies in Design*		
Virtual Reality (VR)	Design Visualization	“We use VR to visualize and simulate design options, enabling stakeholders to experience the space before it is built.” [P9]
	Pain Management	“VR provides immersive distractions for children during painful procedures, reducing their anxiety.” [P10]
Artificial Intelligence (AI)	Clinical Decision Support	“AI-driven systems can help in maintaining accurate patient records and support clinical decision-making.” [P7]
Telemedicine	Remote Care	“Telemedicine is enabling remote consultations, which ensures timely access to care, especially in rural areas.” [P4]
*RQ4: Future Directions in Pediatric Healthcare Design*		
Public Health Approach	Community Well-being	“A public health approach ensures that the design of healthcare facilities not only treats illness but also promotes overall community well-being.” [P3]
Continuous Feedback	Post-Occupancy Evaluations	“Conducting evaluations after the space is in use ensures that the design remains relevant and effective.” [P5]
Culturally Sensitive and Emotionally Engaging Design	Cultural Sensitivity	“Design elements that resonate with patients’ cultural backgrounds can enhance their overall experience and reduce stress.” [P5]
Patient Autonomy	Control Over Environment	“Allowing patients to control aspects of their environment, such as lighting and temperature, improves their comfort and satisfaction.” [P12]

In this study, barriers are defined as systemic or structural constraints, such as privacy concerns, operational inefficiencies, or infection control requirements. Challenges, on the other hand, are process-oriented or interpersonal difficulties, such as engaging diverse stakeholders or addressing the specific needs of neurodivergent patients. This distinction provides a framework for understanding the multifaceted obstacles identified by experts.

**RQ1:** Evolution in Pediatric Healthcare Design and Current Trends

Experts emphasized the shift towards family-centered care and the importance of involving families in the healing process. The need for adaptable and flexible design strategies was highlighted to accommodate changing needs and technologies. Innovations in design, including therapeutic elements and inclusive practices, were considered essential for enhancing patient comfort and engagement. Sustainable and biophilic design practices, which integrate natural elements to promote physical and psychological well-being, were recognized for their positive impact on patient well-being.

“Family-centered care, paired with adaptable design, is essential for creating pediatric environments that can flexibly support diverse and evolving needs of children and families” (P15, Hospital Owner).

**RQ2:** Barriers and Challenges in Implementing Design Strategies

Experts identified barriers such as operational inefficiencies from poor layout designs and privacy constraints in balancing patient supervision with acoustic and visual privacy. Process-oriented challenges included engaging patients and families in the design process and addressing the sensory needs of neurodivergent individuals. These findings underscore the need for systemic solutions alongside participatory approaches to improve design outcomes.

“Balancing privacy with supervision remains a major design challenge in pediatric settings” (P12, Pediatric Healthcare Architect & Researcher).

Systemic barriers such as layout inefficiencies and privacy constraints must be addressed in tandem with participatory processes to improve pediatric care outcomes.

*Systemic barriers such as layout inefficiencies and privacy constraints must be addressed in tandem with participatory processes to improve pediatric care outcomes.*

**RQ3:** Integration of Emerging Technologies

The integration of emerging technologies such as VR and AI was identified as a key factor in transforming pediatric healthcare design. VR is currently used for design visualization and enhancing patient engagement, while AI supports clinical decision-making and improving operational efficiency. Table 3 shows how experts foresee the practical application of these technologies in healthcare environments. Telemedicine was noted for its role in providing remote consultations and timely medical advice.

“VR and AI are driving innovation in pediatric design by enhancing patient engagement and operational efficiency” (P11, Pediatric Healthcare Architect & Researcher).

**RQ4:** Future Directions in Pediatric Healthcare Design

Future directions include addressing systemic barriers, such as operational inefficiencies, while adopting a public health approach to design that promotes community well-being. Continuous feedback mechanisms, such as post-occupancy evaluations, were emphasized as critical to refining design elements. Incorporating culturally relevant and emotionally engaging design elements was highlighted to enhance patient experience, along with providing patients with greater control over their environment to improve autonomy and satisfaction (Figure 2).

“Culturally and emotionally supportive design enhances patient experience and well-being” (P13, Hospital Owner).

While core priorities such as operational efficiency, family engagement, and emerging technologies were widely shared, the emphasis placed on specific design challenges and strategies varied by professional group.

### Analysis of Pediatric Healthcare Design Insights by Professional Role

This analysis (Table 4) highlights the unique contributions, focus areas, and challenges identified by different professional groups in pediatric healthcare design.

**Table 4.** Analysis of Themes in Pediatric Healthcare Design by Professional Group.

Group	Key themes	Specific focus areas	Challenges	Representative quotes
Pediatric Registered Nurse and Researcher	Patient-centered care, emotional support, behavioral health needs, family engagement	Enhancing patient and family experiences, integrating clinical insights into design, addressing emotional and psychological needs of patients	Balancing clinical duties with research, ensuring evidence-based design changes, privacy concerns	*“Our goal is to create spaces where children feel safe, and families are actively involved in the healing process. This means integrating clinical insights into every design decision.” [P3]*
Architect and Researcher	Innovative design, sustainability, sensory and environmental considerations	Integrating research findings into design, creating adaptable and flexible spaces, implementing sustainable practices	Bridging the gap between research and practical application, convincing stakeholders, ensuring designs meet functional and esthetic needs	*“Sustainable design isn't just about materials; it's about creating spaces that adapt to future needs and reduce overall environmental impact.” [P5]*
Pediatric Healthcare Architect	Pediatric needs, comfort, functional design	Designing child-friendly environments, ensuring safety and functionality, incorporating feedback from children and families	Balancing esthetic and functional requirements, addressing diverse needs of pediatric patients, managing budget constraints	*“Our designs aim to create spaces where children feel comfortable and engaged, which in turn supports their healing process.” [P7]*
Pediatric Healthcare Architect and Researcher	Facility design, patient care, adaptability	Creating versatile and flexible spaces, integrating clinical and architectural insights, enhancing patient care through design	Balancing clinical requirements with design innovation, ensuring designs are adaptable, collaborating effectively with clinical staff	*“Our aim is to design healthcare environments that are not only beautiful but also highly functional and adaptable to future changes.” [P11]*
Psychologist and Pediatrics Researcher	Participatory design, engagement, emotional and social support	Involving children and families in design, addressing psychological and emotional needs, using design to facilitate healing	Ensuring meaningful participation, integrating psychological insights into design, managing expectations of stakeholders	*“Participatory design isn't just about asking for feedback; it's about truly integrating the needs and voices of patients and families into the design.” [P10]*
Hospital Owner	Strategic design decisions, operational efficiency, patient experience	Overseeing design projects, making strategic decisions impacting operations, enhancing patient and family satisfaction	Struggles with adapting spaces to changing healthcare demands without extensive redesign. Managing budget and resources, aligning design with strategic goals, ensuring practical and sustainable designs	*“Our focus is on creating healthcare environments that support our operational goals while enhancing the patient experience. We have tried to design a room that will accommodate most patients…* *we know every room can't be a PICU or a NICU room, but we want it to accommodate as many different acuities and needs as possible in one room.” [P13]*
Child Life Specialist	Therapeutic spaces, play and learning, emotional support	Creating therapeutic spaces, incorporating play into healing, supporting emotional needs of children	Ensuring play spaces are safe and functional, integrating therapeutic activities into design, balancing needs of different age groups	*“Therapeutic and play spaces are essential in pediatric healthcare environments, helping children to heal and cope with their hospital experience.” [P14]*

Pediatric nurses and researchers emphasized family engagement and the integration of emotional support within clinical workflows. Their insights highlighted the dual need to address both physical and emotional well-being in pediatric environments. Architects and researchers focused on innovative and sustainable solutions, emphasizing adaptable spaces and biophilic principles to improve patient outcomes while balancing systemic constraints. Pediatric healthcare architects concentrated on child-friendly environments that balance esthetics with functionality, ensuring designs are both engaging and practical for young patients. Psychologists and researchers underscored participatory design and emotional support, advocating for the meaningful involvement of children and families in the design process to align spaces with user needs. Hospital owners prioritized operational efficiency and strategic design decisions to enhance patient experiences while addressing institutional goals. Child life specialists emphasized the importance of therapeutic spaces and play environments in supporting emotional and psychological recovery, particularly through sensory-friendly and engaging designs.

The profession-specific insights highlight the need to bridge operational priorities with emotional and developmental needs in pediatric healthcare environments. A collaborative, interdisciplinary approach is critical to designing spaces that are adaptable, inclusive, and supportive of both clinical outcomes and patient well-being.

## Discussion

Findings from this study highlight expert perspectives on key priorities in pediatric healthcare design, including adaptable and flexible environments, integration of emerging technologies, and culturally and emotionally supportive spaces, for positive patient and family experiences. Experts identified a growing shift toward family-centered care, biophilic and inclusive design, and the use of emerging technologies such as VR and AI to enhance healthcare outcomes (Ahmadpour et al., 2024; Yu et al., 2024). However, significant implementation barriers persist, particularly concerning privacy, infection control, and financial constraints, including systemic resource limitations that affect the delivery of pediatric care (Babbu & Haque, 2023; Kucukkaya et al., 2025). These insights contribute to the ongoing discourse on how pediatric healthcare environments can balance clinical efficiency with patient-centered principles, reinforcing the need for interdisciplinary collaboration between architects, clinicians, and administrators to ensure holistic, evidence-based design solutions.

### Adaptability and Family-Centered Design

A key theme identified was the increasing emphasis on flexible and adaptable environments, which allow healthcare facilities to accommodate shifting patient demographics, evolving medical technologies, and regulatory changes. The study findings highlight the importance of reconfigurable spaces that can be modified based on patient needs, aligning with universal design principles that support diverse populations, including specialized equipment requirements (Nourmusavi Nasab et al., 2020). Implementing adaptable designs in pediatric healthcare remains complex, requiring coordination across operational, technical, and user-centered priorities, often constrained by organizational protocols and budget limitations (Kipps & Erdman, 2025). This finding supports prior research demonstrating that while flexible designs enhance patient experience and staff efficiency, they require strong institutional commitment for successful execution (Halawa et al., 2020).

Family-centered care and flexible design strategies are essential to creating pediatric environments that adapt to changing clinical, developmental, and technological needs.

*Family-centered care and flexible design strategies are essential to creating pediatric environments that adapt to changing clinical, developmental, and technological needs.*

Embedding family-centered care spatially within pediatric hospital environments emerged as a key recommendation, highlighting the need to align family engagement strategies with clinical workflows. The benefits of family-centered care are well documented, including improved patient satisfaction, reduced stress, and better clinical outcomes (Hodgson et al., 2024). However, findings from this study suggest that implementation remains inconsistent due to spatial, cultural, and operational constraints. Prior literature indicates that family-centered design features such as family sleep accommodations, communal spaces, and interactive patient rooms support caregiver engagement, but these features must be effectively integrated into clinical workflows to avoid disruptions (Okoniewski et al., 2022). The findings reinforce the importance of designing spaces that allow families to participate in care while maintaining a balance between privacy and supervision.

### Integration of Emerging Technologies

The adoption of VR, AI, and telemedicine emerged as a transformative opportunity in pediatric healthcare design. The study findings reinforce how VR is increasingly valued for its roles in immersive design visualization, therapeutic applications, and patient engagement, aligning with prior research demonstrating its benefits for alleviating anxiety and supporting pediatric care experiences (Eijlers et al., 2019; Jansen et al., 2023). AI was identified as a critical tool for optimizing clinical decision-making, streamlining workflows, and personalizing care, reinforcing broader trends toward technology-enabled healthcare delivery (Pallavicini & Bouchard, 2019; Topol, 2019). Despite enthusiasm for emerging technologies, the findings underscore persistent challenges related to privacy, ethical concerns, and high implementation costs, which continue to limit broader adoption (Fustino et al., 2019; Montreuil et al., 2021). Successful integration requires interdisciplinary collaboration among designers, healthcare professionals, and technologists to ensure their effective, ethical, and sustainable implementation (Bosch & Lorusso, 2019). One significant barrier to technology integration is the lack of spatial readiness in existing hospital infrastructures.

Integrating technologies like VR and AI into pediatric environments demands alignment with developmental appropriateness, emotional safety, and workflow integration.

*Integrating technologies like VR and AI into pediatric environments demands alignment with developmental appropriateness, emotional safety, and workflow integration.*

While telemedicine has expanded access to pediatric critical care, designing hybrid physical-digital consultation spaces remains challenging due to infrastructure demands, care coordination needs, and variations in implementation outcomes (O’Brien et al., 2024). The need for flexible consultation rooms equipped with integrated digital infrastructure emerged as a significant design priority, reflecting the growing role of hybrid physical-digital healthcare delivery models. Research suggests that while hybrid models improve healthcare accessibility, they require spatial and technological investment to ensure user-friendly implementation (Chen et al., 2020). Future pediatric healthcare environments must proactively incorporate these technologies into early-stage design processes to maximize their benefits.

### Balancing Infection Control, Privacy, and Sensory Considerations

Privacy concerns, operational efficiency, and infection control remain significant barriers to implementing patient-centered and participatory design strategies. Balancing patient privacy with supervision, particularly in open or shared spaces, emerged as a critical and complex design challenge, with experts highlighting the trade-offs between visibility, acoustic control, and emotional well-being (Yu et al., 2024). Prior research suggests that adolescents and neurodivergent patients require greater control over environmental factors, including lighting, noise levels, and spatial boundaries, reinforcing the importance of sensory-friendly and customizable spaces (Higuera-Trujillo et al., 2020). Solutions such as sound-absorbing materials, strategic partitioning, and advanced ventilation systems are essential to maintaining privacy while optimizing workflow and ensuring air quality (Litwin et al., 2023). Adjustable lighting, temperature control, and sensory design elements—including tactile-friendly surfaces and biophilic motifs—can enhance patient comfort and promote emotional and psychological well-being (Shepley et al., 2016). The prominence of Participatory Design in expert perspectives reinforces the need for early and continuous engagement of patients, families, and healthcare professionals to create inclusive, adaptable healthcare environments. Prior studies confirm that early stakeholder engagement improves the usability and effectiveness of pediatric healthcare spaces, leading to more responsive and patient-centered environments (Coyne & Carter, 2018; Larsson et al., 2018). Infection control protocols, while necessary for patient safety, often conflict with emotional and social needs. Single-patient rooms reduce hospital-acquired infections, yet they also limit opportunities for social interaction, increase caregiver burden, and contribute to patient isolation (Sathyanarayanan et al., 2025). While supporting private patient rooms for infection control, experts advocated complementary design strategies that also foster social interaction, emotional support, and flexibility within pediatric environments (Ulrich et al., 2020). Strategies such as biophilic design, smart air filtration, and modular partitions could provide a more holistic approach, balancing clinical safety with social engagement and emotional comfort.

### Culturally Sensitive and Emotionally Supportive Environments

Culturally sensitive and emotionally supportive design emerged as central priorities in shaping pediatric healthcare environments that promote belonging, reduce stress, and support recovery. Research confirms that incorporating cultural elements into hospital design can reduce patient stress, foster a sense of belonging, and improve health outcomes (Kaihlanen et al., 2019; Lauwers et al., 2024). Healthcare environments that reflect patients’ cultural backgrounds through design motifs, language accessibility, and familiar spatial layouts can contribute to a more inclusive and welcoming experience. Culturally responsive spaces should go beyond language-inclusive signage to integrate storytelling areas, community gathering spaces, and interactive design elements that reflect the diverse identities of pediatric patients and their families. Prior research suggests that nature-inspired motifs, social interaction areas, and play-driven engagement zones can reduce anxiety and stress in pediatric patients, reinforcing the therapeutic value of emotionally supportive environments (Higuera-Trujillo et al., 2020; Jiang, 2020). Neurodivergent children and those with mental or behavioral health needs require tailored design approaches. Emphasizing the importance of sensory-friendly environments, the findings highlight the need to prioritize noise control, lighting adjustments, and tactile considerations to create more inclusive spaces for children with sensory sensitivities (Shepley et al., 2016). Biophilic design, which includes access to natural light, greenery, and calming color schemes, has been shown to enhance patient recovery and overall well-being (Litwin et al., 2023). Pediatric hospitals should integrate flexible and patient-controlled settings that allow children to personalize their surroundings, reinforcing autonomy and comfort.

Designing for neurodivergent and behaviorally complex pediatric populations requires inclusive, sensory-responsive, and customizable spatial strategies.

*Designing for neurodivergent and behaviorally complex pediatric populations requires inclusive, sensory-responsive, and customizable spatial strategies.*

These findings align with broader research advocating patient-centered care and participatory design principles in pediatric healthcare environments. The emphasis on flexibility, technology integration, and inclusive design reflects current shifts in healthcare design research, which recognize the need for dynamic and adaptable spaces that support both clinical needs and patient well-being. This study extends existing knowledge by highlighting expert perspectives on emerging design approaches, particularly how cultural sensitivity, sensory integration, and participatory strategies can enhance pediatric healthcare spaces.

### Limitations

This study involved experts primarily from the United States, potentially limiting the diversity of perspectives as cultural and regional factors influence applicability. Experts’ professional backgrounds may have introduced bias, and reliance on self-reported data limits objectivity. The exclusion of direct input from patients or families restricts insights into their experiences and preferences. While focusing on VR and AI, the study offered limited exploration of other technologies, such as telemedicine, which could enhance healthcare design insights. Thematic analysis by a single coder may be a limitation. To address this, iterative re-coding and reflexive practices were applied to reduce bias, supported by detailed documentation to ensure transparency and data-driven findings. The absence of formal inter-coder reliability may reduce interpretive variability achieved through collaborative coding. While the interviews provided rich qualitative insight into priorities and strategies across professional roles, the format did not elicit direct trade-off decisions or explicit recommendations on what to de-prioritize.

### Recommendations for Future Research

Future research should include pediatric patients and families to capture their needs and preferences in healthcare environments. Their direct input offers perspectives not covered in this study. Expanding research across cultures and regions would provide a more comprehensive understanding of how cultural and regional factors influence design preferences and outcomes. A mixed-methods approach, integrating quantitative and qualitative methods, could enrich studies by examining design impacts on recovery times, anxiety levels, and satisfaction. Longitudinal studies should assess the long-term effectiveness of adaptable designs and emerging technologies such as AI, VR, and telemedicine. Research should also focus on balancing infection control with patient-centered design by exploring strategies like modular partitions, hybrid private-social spaces, and smart ventilation systems. Further studies on design strategies for neurodivergent populations, including children with autism and developmental disorders, are needed to create inclusive and supportive healthcare environments. Cost-benefit analyses should evaluate the financial feasibility of adaptable spaces and technology-driven patient engagement tools to inform healthcare investment decisions. Future research could incorporate structured decision-making methods, such as conjoint analysis or discrete choice experiments, to examine how experts prioritize among competing design considerations under resource and operational constraints.

## Conclusion

This study provides a comprehensive examination of pediatric healthcare design, synthesizing expert perspectives to identify current trends, systemic barriers, and actionable pathways for future innovation. By integrating insights from architects, pediatric nurses, psychologists, and healthcare administrators, it highlights the value of interdisciplinary collaboration in creating adaptable and inclusive healthcare environments tailored to the needs of diverse populations. The findings emphasize the critical role of flexible and culturally sensitive design strategies that respond to technological advancements, shifting demographics, and the specific needs of neurodivergent children and vulnerable populations. Emerging technologies, such as VR and AI, are positioned as transformative tools for enhancing patient engagement and operational workflows, provided their integration addresses challenges like privacy and ethical concerns.

This research uniquely contributes to the field by emphasizing participatory design methods, sensory-friendly environments, and the need for expert-driven solutions to systemic challenges like operational inefficiencies and infection control. It underscores the importance of tailoring design features to individual patient needs, including sensory and emotional considerations, as a foundation for future healthcare environments. Future research should integrate patient and family perspectives to complement expert insights, ensuring that healthcare environments holistically address lived experiences alongside systemic requirements. Expanding research across cultural and regional contexts will further enrich the understanding of pediatric healthcare design and its role in supporting community well-being. Actionable recommendations for healthcare designers and policymakers include fostering interdisciplinary collaboration, leveraging emerging technologies responsibly, and prioritizing inclusivity to create environments that comprehensively support young patients and their families.

## Implications for Practice

- *Flexible Design Approaches*: Emphasizing adaptable design solutions is essential to accommodate technological advancements and shifting patient demographics, supporting the long-term functionality of pediatric healthcare facilities.
- *Integration of Emerging Technologies*: Thoughtful incorporation of technologies like VR and AI in the design process and patient care can enhance patient engagement, improve workflow efficiency, and support positive health outcomes.
- *Balancing Privacy With Operational Efficiency*: Addressing privacy concerns while maintaining efficient healthcare operations is crucial, necessitating design strategies that protect patient confidentiality without compromising visibility and supervision.
- *Culturally Sensitive and Emotionally Supportive Environments*: Creating healthcare spaces that reflect the cultural backgrounds and emotional needs of patients and families can reduce stress, promote belonging, and improve overall patient experience and satisfaction.
- *Interdisciplinary Collaboration in Design*: Engaging a multidisciplinary team—including healthcare professionals, designers, patients, and families—in the design process can result in environments that meet the complex and diverse needs of pediatric populations.

## Supplemental Material

sj-docx-1-her-10.1177_19375867251353733 - Supplemental material for From Challenges to Innovations: Expert Insights in Pediatric Healthcare Design Supplemental material, sj-docx-1-her-10.1177_19375867251353733 for From Challenges to Innovations: Expert Insights in Pediatric Healthcare Design by Haripriya Sathyanarayanan and Luisa Caldas in HERD: Health Environments Research & Design Journal
