# Supplementary material for: From Challenges to Innovations: Expert Insights in Pediatric Healthcare Design
Source: HERD. 2025 Jul 24;18(4):180–98. doi: 10.1177/19375867251353733 (PMC12460912; doi:10.1177/19375867251353733)
Supplement: sj-docx-1-her-10.1177_19375867251353733 - Supplemental material for From Challenges to Innovations: Expert Insights in Pediatric Healthcare Design [file sj-docx-1-her-10.1177_19375867251353733.docx]

**Appendix 1**

**Interview Guide: Topics and Sequence**

| Topic | Key Questions |
| --- | --- |
| Design and Trends – Patient Room | 1. Describe recent trends in the architectural design of patient rooms in children’s hospitals?  2. Have you observed any shifts in patient room layout? What factors drive these changes?  3. How has Evidence-Based Design (EBD) influenced the spatial and environmental design of pediatric patient rooms over the past 20 years?  4. Considering the focus on ambient environments in children's hospitals, what are your thoughts?  5. What areas or gaps in evidence do you believe require further research in pediatric patient room design? |
| Patient-Centered Design | 1. How do you consider the needs of young patients in your designs to support their healing process?  2. How do you address patient-centered design,  3 What strategies do you employ to enhance the patient experience?  4. Are there specific metrics or measures you use in the design process for patient experience? |
| Collaborative/Participatory Design | 1. Do you see any value in involving hospitalized children and their families in the design process?  2. How is this involvement typically implemented in current practices?  3. At what stage do you find their input most valuable?  4. In your view, does the current design process empower patients within facility design?  5. How do you ensure the inclusion of family needs in the design process?  6. Given the diversity in a children's hospital population, how do you cater to the broad range of needs?  7. What are the potential benefits and pitfalls of having patients and parents in a participatory versus collaborative role in design?  8. To what extent do architects consider feedback from these collaborative experiences? |
| Immersive Technology | 1. How do you incorporate virtual reality or other immersive technologies in your design process?  2. What role do immersive technologies play in facilitating collaboration within your design team or with stakeholders? |
| Future Directions | 1. What are the most exciting prospects you foresee in the future of healthcare design?  2. How do you envision the role of patient and family engagement in the design of healthcare spaces impacting patient experiences?  3. What methods might be most effective for engaging patients and families in the design process?  4. How could these methods impact spatial and environmental design?  5. What changes do you anticipate in the design workflow and process due to these engagements? |
